# Oxidative Stress and **β**-Thalassemic Erythroid Cells behind the Molecular Defect

**DOI:** 10.1155/2013/985210

**Published:** 2013-09-24

**Authors:** Lucia De Franceschi, Mariarita Bertoldi, Alessandro Matte, Sara Santos Franco, Antonella Pantaleo, Emanuela Ferru, Franco Turrini

**Affiliations:** ^1^Department of Medicine, Section of Internal Medicine, University of Verona, Policlinico GB Rossi, 37134 Verona, Italy; ^2^Department of Life and Reproduction, Section of Biochemistry, University of Verona, 37134 Verona, Italy; ^3^Department of Biomedical Science, University of Sassari, 07100 Sassari, Italy; ^4^Department of Oncology, University of Torino, 10010 Torino, Italy

## Abstract

**β**-thalassemia is a worldwide distributed monogenic red cell disorder, characterized by the absence or reduced **β**-globin chain synthesis. Despite the extensive knowledge of the molecular defects causing **β**-thalassemia, less is known about the mechanisms responsible for the associated ineffective erythropoiesis and reduced red cell survival, which sustain anemia of **β**-thalassemia. The unbalance of alpha-gamma chain and the presence of pathological free iron promote a severe red cell membrane oxidative stress, which results in abnormal **β**-thalassemic red cell features. These cells are precociously removed by the macrophage system through two mechanisms: the removal of phosphatidylserine positive cells and through the natural occurring antibody produced against the abnormally clustered membrane protein band 3. In the present review we will discuss the changes in **β**-thalassemic red cell homeostasis related to the oxidative stress and its connection with production of microparticles and with malaria infection. The reactive oxygen species (ROS) are also involved in ineffective erythropoiesis of **β**-thalassemia through still partially known pathways. Novel cytoprotective systems such as ASHP, eIF2**α**, and peroxiredoxin-2 have been suggested to be important against ROS in **β**-thalassemic erythropoiesis. Finally, we will discuss the results of the major *in vitro* and *in vivo* studies with antioxidants in **β**-thalassemia.

## 1. Introduction

The World Health Organization has identified the hereditary red cell disorders as emerging diseases with high impact on public health systems in both Western and developing countries. Approximately 7% of the global population is carrier of such disorders, and 300,000–400,000 babies with severe forms of these diseases are born each year [1–3]. Severe hereditary hemoglobin disorders of red cells occur at highest frequency in tropical regions, but pop0ulation migrations have ensured that they are present and growing in prevalence in most Western countries. Hemoglobinopathies constitute the single most common monogenic defect worldwide, and among hemoglobin disorders, the thalassemias prominently contribute to [1–4]. *β*-thalassemias (*β*-thal) are characterized by the presence of mutations on beta-globin gene resulting in the absence or reduced synthesis of *β*-globin chains. This is responsible for unbalance in globin chain synthesis with unpaired *α*-chains aggregation. Despite the extensive knowledge of the molecular defects causing *β*-thalassemia, less is known about the mechanisms responsible for the associated ineffective erythropoiesis and reduced red cell survival, which sustain anemia of *β*-thalassemia [5].

## 2. ***β***-Thalassemic Red Cells and Oxidative Stress

**Figure 1:**
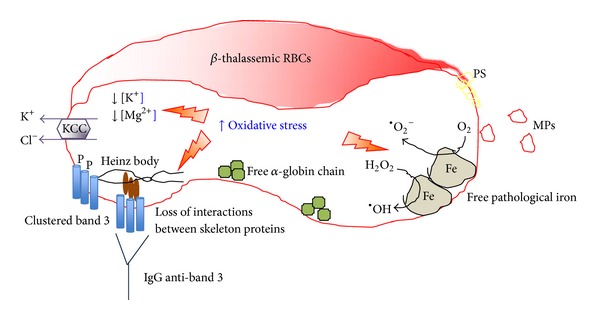
Schematic diagram of abnormalities observed in *β*-thalassemic red cells. The presence of pathological free iron (Fe) close to the membrane is involved in the Fenton reaction producing reactive oxygen species (ROS, ^•^O_2_^−^) contributing to the prooxidant environment of *β*-thalassemic red cells. The unbalance in *α*/*β* chain synthesis results in aggregation of highly oxidative *α* chains. The prooxidant environment is responsible for protein and lipid oxidative damage favoring abnormal clusterization of red cell membrane proteins such as band 3, promoting band 3 tyrosine phosphorylation (P) and exposure of phosphatidylserine (PS). The abnormally clustered band 3 is recognized by naturally occurring anti-band 3 antibody (IgG). The severely damaged *β*-thalassemic red cells released microparticles (MPs). The *β*-thalassemic red cells have short lifespan and are removed by macrophages of the reticuloendothelial systems through PS exposure and IgG anti-band 3 mediated mechanisms. The oxidative stress abnormally activates the K–Cl cotransport (KCC), which promotes K^+^, Cl^−^, and water loss contributing to the reduced red cell K^+^ content that characterizes *β*-thalassemic red cells.

*β*-thalassemic red cells are characterized by extensive red cell membrane oxidative damage related to the excess of unpaired chains, resulting in coclustering of denatured globin and band 3 protein with the generation of large membrane aggregates [6] and decreased membrane thiols [7]. The decompartmentalization of cellular free iron and its nonrandom association to the hemichrome band 3 aggregates [8] observed in *β*-thal red cells further amplifies the oxidative environment of *β*-thal erythrocytes [9]. These small amounts of pathological free iron from unpaired hemoglobin chains could initiate self-amplifying redox reactions that simultaneously deplete cellular reduction potential, oxidize additional hemoglobin, and trigger phosphorylative responses initiating membrane destabilization [10–12], accelerating the red cell blood destruction (Figure 1). The membrane–damaging effects of unpaired chains have been also demonstrated by entrapping hemoglobin chains in normal erythrocytes [13].

Studies in *β*-thal erythrocytes have shown that proteins from both cytoskeleton network and membrane are targeted by the oxidative stress. In red cells spectrins are key proteins of the cytoskeleton network, [14, 15]. Studies in *β*-thal erythrocytes show that spectrins are involved by the oxidative damage, resulting in perturbation of their interactions with other cytoskeleton proteins such as actin or with proteins from multiprotein complexes bridging the membrane to the cytoskeleton as protein 4.1 [15]. In *β*-thal red cells, the loss of the stability between the cytoskeleton network and the junctional multiprotein complexes might favor the abnormal clusterization of transmembrane protein such as band 3 (Figure 1). In particular, two cysteine residues located in the cytoplasmic domain of band 3 show a peculiar reactivity to oxidants being 10-fold more reactive than GSH (Ferru E. and Pantaleo A., personal communication). This uncommon reactivity appears to be finalized to the regulation of band 3 tyrosine phosphorylation in anchoring of the membrane cytoskeleton to the lipid bilayer [16–18]. This function is linked specifically to its association with adducin and ankyrin in two distinct junctional complexes [19]. Rupture of either of these two bridges yields an erythrocyte that spontaneously loses membrane surface through vesiculation/blebbing. Recent studies from our lab demonstrate that Syk-mediated tyrosine phosphorylation of oxidatively modified band 3 leads to complete inhibition of ankyrin binding and the consequent dissociation of band 3 from the cytoskeleton [20]. When red cells are mechanically stressed, they bleb membrane surface and vesiculate. Indeed, in scrutinizing the literature, we have noted that membrane vesiculation and release of circulating microparticles (MPs) constitute a common characteristic of erythrocyte pathologies (sickle cell disease, G6PDH deficiency, *β*-thalassemia) that are characterized by elevated band 3 tyrosine phosphorylation. A study showed significantly higher levels of circulating MPs originated from red cell membranes in *β*-thalassemia intermedia patients compared to controls, especially in splenectomized patients [21]. MPs originating from red cell membranes are also considered a major cause of premature atherosclerosis described in thalassemia intermedia patients [22]. 

Band 3 tyrosine phosphorylation observed in *β*-thalassemia may impact additional erythrocyte functions. Band 3 organizes a complex of glycolytic enzymes on the membrane and thereby controls the flux of glucose between the pentose phosphate pathway (PPP) and glycolysis. Phosphorylation of band 3 by Syk leads to displacement of these glycolytic enzymes from an inhibitory site on band 3, resulting in activation of glycolysis and a decline in red cell reducing power through NADPH production [23]. 

Several lines of evidence indicate that in thalassemias and in unstable hemoglobin diseases, damaged red cells are removed by spleen through an immunological mechanism [6, 24]. High amounts of anti-band 3 antibodies (NAbs) and C3b are constantly found bound to band 3-hemichromes aggregates [6], leading to intense phagocytosis. Those high molecular weight complexes containing IgG and C3b were isolated from red cell membranes in thalassemic, sickle cell, and haemoglobin C patients [25, 26]. The colocalization of the various components was also demonstrated by fluorescence microscopy [26]. Interestingly, anti-band 3 NAbs eluted from thalassemic red cells recognize dimeric/oxidized band 3 [6]. In thalassemias, a correlation has been found between the degree of anemia and the amount of anti-band 3 NAbs and of band 3/hemichrome copolymers in red cell membranes indicating a central role of NAbs in *β*-thal red cells removal [24]. In conclusion, the most relevant membrane changes linked to hemolysis and complications in thalassemias appear to deal with the binding of naturally occurring antibodies and with the destabilization of the red cell membrane leading to membrane loss and microparticle release.

## 3. The Membrane Oxidative Damage Participates to the Removal of ***β***-Thal Red Cells by the Macrophage System: The Connection with Malaria Infection

Previous studies indicate a major role of immune determinants in the removal of *β* thalassemic red cells [24]. The interactions between thalassemic red cells and plasmodia appear to play a major role in natural and acquired protection to malaria. Heterozygous *α*- and *β*-thalassemias are extremely frequent in malaria endemic areas displaying a well-balanced hematological situation [27], while there is a widespread consensus that thalassemias determine an efficient resistance to severe malaria [28]. In particular, *α*-thalassemias are the most common mutation in malaria endemic regions and are considered to confer protection against clinical manifestations related to both severe forms [29–32] or uncomplicated malaria [33]. Although the molecular basis of the mechanism of resistance is not completely understood it has been observed that *α*-thalassemic red cells infected with *P. falciparum* bind higher amounts of antibodies or complement factors from immune sera [34, 35]. Moreover, lower levels of the complement receptor-1 (CR1) have been found in *α*-thalassemic red cells, and a reduction of infected red cells to form rosettes (associated to severe malaria) has been associated to CR1 deficiency [36].

Heterozygous *β*-thalassemia also confers protection against severe malaria and uncomplicated malaria in children [35, 37]. One study indicates that heterozygous *β*-thalassemic red cells are unable to sustain the normal development of *P. falciparum* “*in vitro*” [38]. In addition, similarly to *α*-thalassemia, immunological determinants appear to be involved in a more efficient recognition of infected *β*-thalassemic red cells [34, 39]. More recently it has been observed that both heterozygous *α*- and *β*-thalassemic red cells do not apparently damage the parasites but induce a loss of viability of the infected erythrocytes and their removal by macrophages [40]. We have also observed that the deletion of 11 amino acids at the band 3 amino terminal (band 3 Neapolis) results in a profound red cell membrane destabilization, in increasing naturally occurring IgG binding, and a reduction of *P. falciparum* to grow in these red cells [41]. These findings suggest that different mutations, including Southeast Asian ovalocytosis, elliptocytosis, and unstable hemoglobins affecting structure, functions, and antigenic properties of the red cell membrane, might interfere with the development of malaria parasites [42–44]. 

In that respect, several studies have identified proteins that are phosphorylated upon malarial infection [41, 45–47]. Band 3 represents the earliest tyrosine phosphorylation event during parasite development, beginning at low levels during early ring stage parasitemia and increasing continuously until parasite egress [48]. We have recently shown that in red cells from heterozygous *β*-thalassemic subjects the process of band 3 phosphorylation is amplified [16], suggesting that band 3 related destabilization of the host red cell membrane may be involved in the mechanism of malaria resistance. In support to this hypothesis, recent data demonstrated that long-lived radicals, indolone-N-oxide derivatives (INODs), exert antiplasmodial activity in the low nanomolar range accelerating the rate of phosphorylation of band 3, its clustering, and altering the stability of the erythrocyte membrane without a direct effect on parasite targets [49]. The relationships between band 3 phosphorylation, its clustering, and the binding of naturally occurring antibodies in malaria and thalassemias remain to be fully established [50].

## 4. ***β***-Thalassemia Red Cells Abnormal Activation of K–Cl Cotransport with K^**+**^ Loss Related to the Membrane Oxidative Damage

The observation of the relationship between hemoglobin precipitation and reduced cell K^+^ content in *β*-thal erythrocytes has suggested a link between red cell membrane oxidative damage and abnormalities of red cell membrane ion transport pathways in *β*-thal red cells [51, 52]. *In vitro* studies with oxidant agents mimicking *β*-thal red cell membrane damage as phenylhydrazine (PHZ) have helped in dissecting the contribution of the oxidative stress in activation of different membrane ion transport pathways involved in generation of red cell with reduced K^+^ content such as in *β*-thalassemia [51, 52]. The red cell membrane transport can be divided into (i) the energy driven systems as the Na–K ATPase pump; (ii) the gradient driven systems as the Na–K–2Cl cotransport and the K–Cl cotransport; (iii) the exchange as the Na–H or the Na–Li exchange; and (v) the channel as the Gardos channel [52–59]. In *β*-thal red cells we have shown that the activity of the K–Cl cotransport (KCC) is increased, and its abnormal activation is related to the severe membrane oxidative damage characterizing *β*-thal erythrocytes [51, 52] (Figure 1). When *β*-thal red cells are treated with DIOA ([(dihydroindenyl)oxy]alkanoic acid), the specific inhibitor of the K–Cl cotransport, the red cell K^+^ content increases supporting the key role of K–Cl cotransport in K^+^ loss of *β*-thal erythrocytes [51, 60]. However, the inhibition of the K–Cl cotransport by DIOA is limited by DIOA toxicity. Studies on K–Cl cotransport in hemoglobinopathies have shown that the activity of the K–Cl cotransport might also be increased by reduced red cell Mg^2+^ content, which characterized *β*-thal red cells [61–63] (Figure 1). Thus, different strategies have been evaluated to pharmacologically inhibit the activity of the K–Cl cotransport in *β*-thal erythrocytes. These target two factors involved in K–Cl cotransport activation: the membrane oxidative damage and the red cell Mg^2+^ content. The development of animal models for *β*-thal mimicking the human counterpart have been crucial to evaluate *in vivo* the pathophysiological events involved in activation of K–Cl cotransport and generation of red cells with low K^+^ content [64–66]. 

Deferiprone, an iron chelator that crosses the red cell membrane and accumulates in erythrocytes, has been shown to ameliorate *β*-thal red cell survival in a mouse model for *β*-thalassemia and to reduce the abnormal activation of K–Cl cotransport in red cells from human *β*-thal patients treated with deferiprone for 3 months [10, 67]. Abnormalities of Mg^2+^ metabolism have been described in *β*-thalassemia and abnormally low red cell Mg content characterized *β*-thal red cells, which contribute to K–Cl cotransport activation [64, 68, 69]. Studies in animal models for *β*-thalassemia have shown that dietary Mg^2+^ supplementation alone or in combination with hydroxyurea can reduce the activity of K–Cl cotransport, increase the red cell K^+^ content, and ameliorate the *β*-thal hemolytic phenotype [62, 64]. In untransfused *β*-thal intermedia patients treated with Mg-pidolate (1.2 mEq/Kg/d), we observed significant increase in red cell Mg^2+^ content, reduction in K–Cl cotransport activity, increase of red cell K^+^ content, and decrease of reticulocyte count [63]. These data suggest that modulation of K–Cl cotransport through different strategies ameliorates the hematological phenotype of both mouse model and human subjects with *β*-thalassemia.

## 5. Novel Cytoprotective Systems and ***β***-Thalassemia

Although *β*-thal red cells have been largely studied in the last decades and the contribution of the oxidative stress has been documented in shortening *β*-thal red cell lifespan, the role of reactive oxygen species (ROS) in ineffective erythropoiesis of *β*-thalassemia has been only partially investigated. 

**Figure 2:**
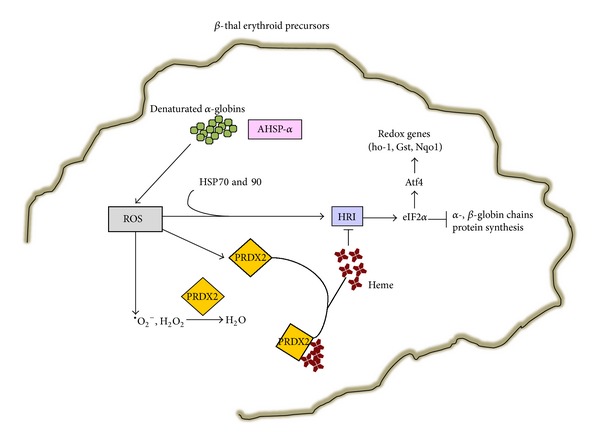
Schematic model of novel cytoprotective mechanisms in response to oxidative stress in *β*-thalassemic (*β*-thal) erythroid precursors. In *β*-thalassemic erythropoiesis the radical oxidative species (ROS) induces peroxiredoxin-2 (PRDX2) expression. In the early stage of *β*-thalassemic erythropoiesis, ROS and heme levels are both increased and PRDX2 acts on both targets; in more mature cells, when ROS levels are still high and heme levels are reduced, ROS might become the PRDX2 major target (see text for details). ROS promotes HRI activation, which requires the heat shock proteins 70 and 90 (HSP70,-90). HRI activation results in phosphorylation of the *α*-subunit of eIF2, an important regulatory translation initiating factor, which inhibits the *α*-, *β*-globin chain synthesis and activates the Atf4 pathway towards redox genes such as heme-oxygenase-1 (ho-1), glutathione S-transferase (gst), and NAD(P)H quinone oxidoreductase 1 (Nqo1). The upregulation of these genes in combination with the decrease in *α*-, *β*-globin chain synthesis might beneficially affect the ineffective erythropoiesis of *β*-thalassemia. The *α* chains (AHSP, *α* hemoglobin-stabilizing protein) is another cytoprotective system, which partially protects the erythroid precursors from the *α* chain excess. AHSP binds free *α*-globin chains, stabilizing their structure. AHSP prevents their precipitation and might be important in *β*-thalassemic erythropoiesis characterized by unbalance in globin chain synthesis.

Previous studies have identified a small protein stabilizing the *α* chains (AHSP, *α* hemoglobin-stabilizing protein) as an important protein facilitating hemoglobin assembly and partially protecting the erythroid precursors from the *α* chain excess (Figure 2). In fact, AHSP binds free *α*-globin chains, stabilizing their structure and prevents their precipitation [77–80]. Indeed, anemia of *β*-thalassemic mice is more severe in *β*-thalassemic/AHSP-deficient mice [77–79]. However, the impact of AHSP deficiency in *β*-thalassemia patients is still under evaluation and the link between decreased AHSP expression and severity of *β*-thalassemic syndromes remains speculative [79].

Another protective factor in *β*-thalassemic erythropoiesis is the heme-regulated inhibitor of protein translation (HRI) that represses globin translation in heme-deficient erythroid precursors [81] (Figure 2). HRI is the heme-regulated eIF2*α* kinase that phosphorylates a subunit of eIF2, a crucial regulatory translational initiating factor. Studies in *in vitro* systems have shown that the activation of HRI involves also ROS and requires the molecular chaperones heat shock proteins 70 and 90 (HSP70 and -90) [82]. Thus, *β*-thalassemic erythropoiesis characterized by ROS and unbalance of globin chain synthesis might be an interesting model to validate the role of HRI-eIF2*α* pathway. In fact, *β*-thal mice genetically lacking HRI show a more severe hematological phenotype compared to *β*-thal mice, supporting the key role of eIF2*α* in stress erythropoiesis [81, 83]. Recently, HRI-dependent eIF2*α*P has been also shown to enhance the translation of the Atf4 in mouse erythroid cell precursors exposed to oxidative stress [81, 84]. This results in upregulation of genes from antioxidant systems such as heme-oxygenase-1 (ho-1), glutathione S-transferase-*μ* (gst*μ*), and NAD(P)H quinone oxidoreductase 1 (Nqo1). In *β*-thal erythroid precursors the increase of eIF2*α*P by salubrinal treatment results in inhibition of globin chain synthesis, suggesting that pharmacological modulation of eIF2*α*P might possibly impact the *β*-thal ineffective erythropoiesis through inhibition of globin chain synthesis and possibly through upregulation of antioxidant systems (Figure 2).

Another novel cytoprotective system recently described in *β*-thalassemia is peroxiredoxin-2 (PRDX2) (Figure 2). PRDX2 is a typical 2-cysteine (Cys51 and -172) peroxiredoxin, which acts as antioxidant and molecular chaperone in different cell types [85, 86].

### 5.1. Peroxiredoxin-2 and *β*-Thalassemia

PRDX2 is the third most abundant cytoplasmic protein in red cells and is able to reduce and detoxify a vast range of organic peroxides, H_2_O_2_, and peroxynitrite [87, 88]. Recently, we have shown that PRDX2 expression is increased in a mouse model for *β*-thalassemia [65, 89]. However, PRDX2 membrane translocation in *β*-thal red cells is reduced despite the severe membrane oxidative damage. Since *β*-thal red cells are characterized by the presence of hemichrome membrane association, we have shown that PRDX2 is displaced by the membrane in function of the proportion of denaturated and oxidized hemoglobin recovered on the membrane as hemichromes [89]. Thus, in *β*-thal red cells PRDX2 is unable to translocate to the membrane in response to oxidative damage since hemichromes mask PRDX2 binding site on the membrane. Based on the evidences that band 3 is the docking site for hemichrome on the red cell membrane [6, 41], we hypothesize that band 3 might be the binding site also for PRDX2. To address this question we have studied the interactions of recombinant PRDX2 with the cytoplasmic domain of band 3 [90]. We show that PRDX2 binds to the cytoplasmic domain of band 3 with different experimental methodological approaches including cross-linking studies, fluorescence and dichroic measurements, surface plasmon resonance analysis, and proteolytic digestion assay. This finding is also supported by the absence of PRDX2 membrane association in a patient with band 3 Neapolis, a truncated isoform of band 3 lacking the N-terminal 11 amino acid residues [41, 90]. We believe that the membrane association of PRDX2 with band 3 might be important in protecting band 3 from oxidative damage and its associated membrane proteins. In the context of *β*-thal red cells the presence of hemichrome masking the docking site for PRDX2 contributes to further amplification of red cell membrane oxidative damage characterizing *β*-thalassemic erythrocytes. 

Looking for novel cytoprotective mechanisms in *β*-thal erythropoiesis we have carried out a classic proteomic analysis of erythroid precursors from healthy and *β*-thal intermedia subjects. We identify PRDX2 as one of the antioxidant systems differently expressed during erythroid maturation in *β*-thal erythroid cells compared to controls [91]. In other cell types PRDX2 has been demonstrated to be induced by oxidative stress and that cells overexpressing PRDX2 are more resistant to the oxidative stress [92, 93]. To evaluate the impact of PRDX2 during erythropoiesis in cells, exposed *in vitro* to oxidative stress, we silenced PRDX2 in K562 cells and we observed decreased differentiation and reduced cell survival, supporting the important role of PRDX2 as cytoprotective system during stress erythropoiesis [91]. Since PRDX2 is highly expressed during *β*-thal erythropoiesis, we speculate that its role might not be limited to antioxidant function. Using recombinant PRDX2 we demonstrate that PRDX2 specifically binds heme with decreased PRDX2 peroxidase activity. In *β*-thal erythropoiesis we propose that in early *β*-thal erythroid precursors, characterized by high levels of ROS and heme, PRDX2 targets both ROS and heme to reduce oxidative stress. While in late *β*-thal erythropoiesis, when ROS levels are still high but heme levels are reduced, ROS might be the major target of PRDX2 (Figure 2) [91]. Future studies using sorted erythroid precursors at different stage of maturation [94] need to be carried out to better characterize the role of PRDX2 in *β*-thal erythropoiesis.

## 6. Antioxidants as Therapeutic Strategy in ***β***-Thalassemia

Since the oxidative stress plays a key role in the pathogenesis of *β*-thalassemia, the use of various molecules with antioxidant properties as possible therapeutic strategy in *β*-thalassemia has been explored (Table 1). A pilot trial with large dose of oral vitamin E, prompted by the abnormally low levels of this vitamin in plasma of patients with *β*-thal intermedia, showed a decrease in the levels of malonylaldehyde but not in transfusion requirements [70, 73]. Amelioration of *β*-thal red cell osmotic fragility has been also reported in *β*-thal major patients treated with vitamin E supplementation [71]. In another study with vitamin E supplementation involving *β*-thal intermedia patients, an improvement of plasma oxidative stress has been reported, supporting the role of vitamin E as antioxidant agent with multitarget effects in *β*-thalassemia [72]. The polyphenol curcumin caused a significant inhibition of lipid peroxidation in *β*-thal red cell ghosts [73] and an improvement in methemoglobin levels in *β*-thal patients treated with curcumin, with no effects on patients hemoglobin levels [74].

Another antioxidant molecule that has been evaluated in *β*-thalassemia is the fermented papaya preparation (FPP). Studies *in vitro* and *in vivo* in both mouse model for *β*-thalassemia and *β*-thal human subjects have shown that FPP reduces the *β*-thal red cell oxidative stress, the membrane lipid peroxidation, and the percentage of PS positive red cells, and increases reduced glutathione (GSH). The amelioration of red cell features induced by FPP is also associated with reduced red cell phagocytic index, suggesting possible reduction in removal of FPP treated *β*-thal red cells from the peripheral circulation by the macrophage system [73, 76]. Recently, in a mouse model for *β*-thal it was shown that a novel semisynthetic flavanoid, 7-monohydroxyethylrutoside (monoHER), reduces the percentage of PS positive cells, increases the red cell membrane and plasma vitamin E content and red cell K^+^ content with beneficial effects on mouse *β*-thal erythropoiesis [66]. The thiol compound N-acetylcysteine amide (AD4), the amide form of N-acetyl cysteine (NAC), has been studied both *in vitro* in *β*-thal red cells and *in vivo* in mouse model for *β*-thalassemia [75]. Amer et al. show that in *β*-thal mouse red cells *in vitro* and *in vivo* AD4 significantly improves GSH levels and reduces the percentage of PS positive cells and the *β*-thal phagocytic index, suggesting that the restoration of thiol levels in *β*-thal red cells might represent an additional strategy to antioxidant treatment in *β*-thalassemia. In a mouse model for *β*-thalassemia we have recently shown that resveratrol, a polyphenolic-stilbene, ameliorates the *β*-thal ineffective erythropoiesis through the activation of FOXO3, transcriptional factor, and reduces the oxidative stress in circulating *β*-thal red cells [95].

## 7. Future Prospective

In conclusion, the oxidative stress plays a central role in the pathogenesis of anemia in *β*-thalassemia. The emerging picture for treatment of *β*-thalassemia is that abnormalities ranging from red cell membrane proteins structure and function and membrane ion transport pathways to novel cytoprotective systems in erythropoiesis might constitute new pharmacological targets for treating *β*-thalassemia. Future studies should be designed to evaluate *in vivo* novel antioxidant strategies with multitarget effects on both mature *β*-thal red cells and erythropoiesis with the final goal to impact anemia of *β*-thalassemia.

## Figures and Tables

**Table 1:** Effects of different antioxidant treatments in *β* thalassemia.

Molecule	Model	Evidences	Ref.
Vitamin E	*β*-thal intermedia patients *(in vivo *study)	↓ MDA Amelioration in the oxidation of low density lipoproteins Amelioration of RBCs osmotic fragility No changes in transfusion requirement	[70–72]
Curcumin	*β*-thal patients *(in vitro *study) *β*-thal/HbE patients *(in vivo *study)	↓ lipid peroxidation ↓ methemoglobin, but no changes in Hb levels	[73, 74]
FPP	*β*-thal major and intermedia patients *(in vitro *study) *β*-thal/HbE patients *(in vivo *study) *β*-thal mouse model *(in vivo) *	↓ ROS ↑ GSH ↓ PS positive RBCs ↓ RBCs phagocytosis No effects on Hb levels	[73, 75]
MonoHER	*β*-thal mouse model *(in vivo) *	↑ RBCs K^+^ content ↓ KCl cotransport activity ↓ PS positive RBCs ↑ RBCs membrane and plasma vitamin E levels Amelioration of *β*-thal mouse erythropoiesis	[66]
AD4	*β*-thal major and intermedia patients *(in vitro *study) *β*-thal mouse model *(in vivo *study)	↓ ROS ↑ GSH ↓ PS positive RBCs ↓ RBCs phagocytosis No effects on Hb levels	[76]

*β*-thal: *β*-thalassemia; MDA: malonylaldehyde; RBC: red blood cell; Hb: hemoglobin; PS: phosphatidylserine; GSH: reduced glutathione peroxidase; ROS: reactive oxygen species; FPP: fermented papaya preparation; AD4: N-acetylcysteine amide.
